# Neuropsychiatric Clues to Herpes Simplex Virus Encephalitis Presenting as Normal Pressure Hydrocephalus

**DOI:** 10.1155/crps/9748747

**Published:** 2026-06-03

**Authors:** Christopher E. Potts, Alana Kassis, Akbar Ali, James Issa, Usman Alizai, Dakota May

**Affiliations:** ^1^ Department of Psychiatry and Behavioral Medicine, Marshall University Joan C. Edwards School of Medicine, Huntington, West Virginia, USA, marshall.edu; ^2^ Department of Cardiac Surgery, The Ohio State University Wexner Medical Center, Columbus, Ohio, USA, osu.edu

## Abstract

Herpes simplex virus type 1 (HSV‐1) encephalitis can mimic neurodegenerative or structural disorders, particularly in older adults presenting with cognitive or behavioral symptoms. We report the case of a 73‐year‐old woman with progressive gait instability, urinary incontinence, and cognitive decline, which was initially attributed to normal pressure hydrocephalus (NPH). Despite transient improvement after two lumbar punctures and neuroimaging suggestive of significant ventriculomegaly, the patient’s symptoms progressed with increasing paranoia, hallucinations, and agitation. Psychiatric consultation worked up the NPH diagnosis and identified various behavioral abnormalities—such as fluctuating delirium and psychosis—inconsistent with classic NPH. After 20 days of admission, restructuring of the differential and extensive infectious and metabolic testing led to the identification of HSV‐1 DNA in cerebrospinal fluid (CSF). The patient improved markedly with prompt acyclovir therapy. This case underscores the diagnostic overlap and mimicry between infectious and neurodegenerative processes and highlights the role of psychiatric input in reframing atypical neurocognitive presentations and expanding the differential of treatment‐resistant NPH in the presence of inconsistent symptomatic progression.

## 1. Introduction

Herpes simplex virus type 1 (HSV‐1) encephalitis is the most common cause of sporadic viral encephalitis in adults and represents a true neurologic emergency requiring prompt recognition and treatment [1]. The disease has an estimated annual incidence of two to four cases per million individuals [2] and is associated with a high untreated mortality rate of ~70%; among survivors, up to 97% fail to recover their baseline level of functioning [1]. Even with treatment, many patients experience lasting neuropsychiatric and cognitive impairments.

HSV‐1 encephalitis results from reactivation of latent virus within the trigeminal ganglia, followed by viral spread to the frontal and temporal lobes, leading to extensive parenchymal inflammation and necrosis [1]. Clinically, the infection typically presents with fever, confusion or disorientation, personality or behavioral changes, altered mental status, seizures, and focal neurological deficits [3]. However, early symptoms often resemble other disorders, especially in older and immunocompromised individuals [1]. This diagnostic ambiguity may delay antiviral therapy and worsen outcomes, emphasizing the importance of early laboratory evaluation and neuroimaging. The most characteristic MRI finding of HSV‐1 encephalitis is asymmetric T2‐weighted hyperintense lesions reflecting edema in the mesiotemporal, orbitofrontal, and insular regions, helping differentiate HSV‐1 from other intracranial pathologies [1].

The clinical manifestations of HSV‐1 encephalitis may overlap with other intracranial disorders, such as normal pressure hydrocephalus (NPH). NPH is a chronic condition characterized by the triad of gait disturbance, urinary incontinence, and cognitive impairment [4]. Although uncommon, few case reports have described urinary incontinence [5] or retention [6] in patients with HSV encephalitis. The cognitive profiles of NPH and HSV‐1 can overlap, complicating the distinction between them. On MRI, NPH typically shows ventricular enlargement with disproportionate widening of the inner cerebrospinal fluid (CSF) spaces and frontal lobe atrophy and may also demonstrate periventricular hyperintensities related to transependymal CSF stagnation or chronic small‐vessel ischemic change. These findings are generally nonspecific and differ from the temporal lobe abnormalities more characteristic of HSV‐1 encephalitis [4].

Because HSV‐1 encephalitis and NPH may share overlapping cognitive and behavioral features, psychiatric consultation is critical for identifying findings inconsistent with structural pathology. This case illustrates how psychiatric assessment can identify clinical features inconsistent with isolated NPH, prompting broader diagnostic reconsideration within the context of potential comorbid neurodegenerative processes and facilitating recognition of HSV‐1 encephalitis.

## 2. Case Presentation

A 73‐year‐old woman with a history of hypertension, hyperlipidemia, and generalized anxiety disorder presented to the emergency department after several weeks of progressively worsening gait disturbance, falls, urinary incontinence, and cognitive decline. Outpatient neuroimaging performed prior to admission demonstrated ventriculomegaly with mild cortical atrophy, raising concern for NPH. The patient’s family reported escalating episodes of confusion, paranoia, and delusions that “someone was breaking into her home.” She had not undergone prior evaluation or treatment for NPH. The patient did not exhibit consistent febrile episodes prior to or during the early course of hospitalization.

Two diagnostic lumbar punctures revealed opening pressures of 17 and 15 cm H_2_O within normal reference ranges, leading to transient improvement in gait stability. These findings, along with urinary incontinence, initially supported an NPH diagnosis. Neurosurgery was consulted for possible ventriculoperitoneal shunt placement, and the patient was admitted. Formal neuropsychological testing was not feasible during the acute phase due to fluctuating delirium; a screening cognitive assessment (MoCA) was obtained, and overall cognitive status was assessed through serial bedside evaluation and psychiatric consultation.

Upon admission, physical and neurologic examinations revealed a shuffling gait, hyperreflexia, bilateral ankle clonus, and impaired short‐term memory without focal motor deficits. MRI of the brain on admission demonstrated generalized cortical atrophy with ventriculomegaly consistent with central volume loss rather than typical NPH and did not show the asymmetric mesial temporal or frontal lobe abnormalities more classically associated with HSV‐1 encephalitis.

**Figure 1:**
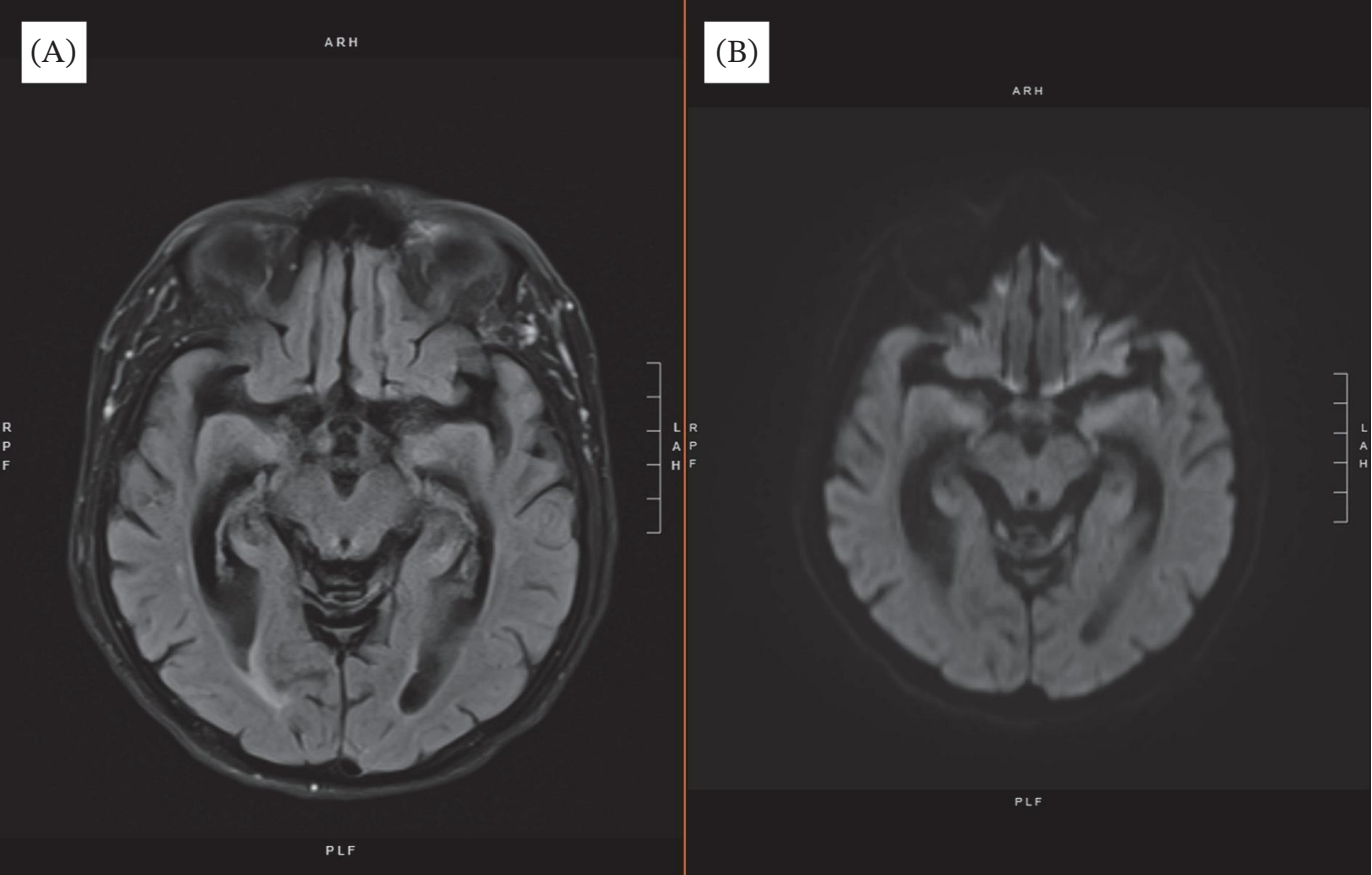
Admission brain MRI without definitive HSV‐1 encephalitis findings. (A) Axial T2‐FLAIR image and (B) axial diffusion‐weighted image (DWI) at the temporal lobe level demonstrate ventriculomegaly without clear mesial temporal, insular, or frontal lobe signal abnormality suggestive of HSV‐1 encephalitis.

**Figure 2:**
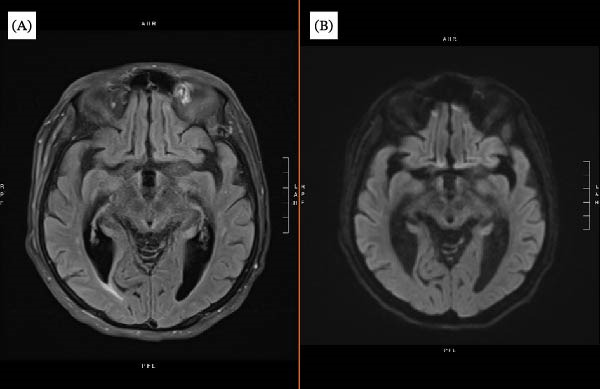
Follow‐up brain MRI demonstrating subtle interval temporal lobe signal abnormality in the setting of CSF‐confirmed HSV‐1 encephalitis. (A) Axial T2‐FLAIR image and (B) axial diffusion‐weighted image (DWI) at the temporal lobe level show subtle interval signal abnormality compared with the admission MRI.

During hospitalization, the patient exhibited worsening delirium, visual hallucinations, and severe agitation, delaying shunt placement for psychiatric workup. Psychiatry noted various discrepancies between presentation and imaging findings, including inconsistent response to CSF drainage, and recommended an expanded infectious evaluation. Subsequent CSF polymerase chain reaction (PCR) testing detected HSV‐1 DNA, confirming viral encephalitis after 20 days of hospitalization. Acyclovir therapy (10 mg/kg every 8 h) was promptly initiated and highly effective. In the next few days, agitation drastically subsided, orientation improved, and urinary control partially returned. Follow‐up MRI demonstrated temporal lobe hyperintensities compatible with HSV‐1 encephalitis, representing interval evolution from the initial MRI, which lacked classic mesial temporal involvement. The patient was discharged to an inpatient rehabilitation facility with significant improvement in agitation, orientation, and urinary control. While gait and cognitive deficits improved, they had not fully returned to baseline at the time of discharge (Figures 1 and 2 and Table 1).

**Table 1:** Clinical timeline of symptom progression, diagnostic evaluation, and treatment course.

Time	Clinical events
∼3–4 months prior	Onset of gait disturbance, cognitive decline, urinary incontinence, paranoia, hallucinations, and frequent falls following a motor vehicle collision.
∼6 weeks prior	First outpatient lumbar puncture; opening pressure 17 cm H_2_O with transient improvement in symptoms for approximately 1 week.
∼3 weeks prior	Second outpatient lumbar puncture; opening pressure 15 cm H_2_O with transient improvement for approximately 7–10 days.
Day 0 (admission)	Presented with worsening gait instability, frequent falls, and altered mental status. CT head showed no acute intracranial process. The patient did not exhibit consistent febrile episodes prior to or during the early hospital course.
Hospital Day 1	Neurosurgical evaluation documented progressive memory loss, urinary incontinence, gait disturbance, hallucinations, and frequent falls. Brain MRI demonstrated ventriculomegaly and atrophy without mesial temporal or frontal lobe abnormalities suggestive of HSV‐1 encephalitis.
Hospital Day 3	Neurology documented a MoCA score of 18/30 before planned lumbar drain trial for suspected normal pressure hydrocephalus.
Hospital Day 6	Acute delirium, paranoia, and agitation developed, complicating continued evaluation for normal pressure hydrocephalus.
Hospital Day 7	Psychiatry was consulted for agitation and aggression. Neurobehavioral findings raised concern for delirium and for a process not fully explained by isolated normal pressure hydrocephalus.
Hospital Day 17–18	HSV‐1 was identified in cerebrospinal fluid, and intravenous acyclovir was initiated. Cerebrospinal fluid from the most recent lumbar puncture did not demonstrate pleocytosis. Orolabial lesions were also noted.
Following antiviral therapy	Agitation, orientation, and urinary control improved. Gait and cognitive deficits also improved, although they had not fully returned to baseline by discharge.
∼3 weeks after initial presentation	Follow‐up brain MRI demonstrated subtle temporal lobe hyperintensities consistent with HSV‐1 encephalitis, representing interval evolution from the initial MRI.
Discharge	Transferred to inpatient rehabilitation with partial clinical improvement.

## 3. Discussion

HSV‐1 encephalitis remains the most common cause of sporadic fatal encephalitis worldwide [7]. Although classically characterized by rapidly progressing fever, focal deficits, seizures, and temporal lobe involvement, many patients present with nonspecific cognitive and behavioral disturbances that can mimic neurodegenerative, structural, or psychiatric conditions [1].

Symptoms of HSV‐1 encephalitis are often attributed to other pathologic processes before a comprehensive immunologic workup can confirm a working diagnosis. In this case, the patient’s initial symptoms and imaging results, such as urinary incontinence, slightly increased pressures on spinal tap, and significant ventriculomegaly, appropriately raised clinical concern for a working diagnosis of NPH, creating a diagnostic pitfall and delaying recognition of the true etiology. The absence of comprehensive CSF analysis during earlier lumbar punctures performed for suspected NPH represents a limitation and may have contributed to delayed diagnosis in this case. This diagnostic delay permits ongoing viral replication, progressive CNS involvement, worsening neuronal injury, and ultimately has a significant impact on clinical outcomes [8].

Although early initiation of acyclovir is strongly associated with improved outcomes in HSV‐1 encephalitis, delayed diagnosis remains common, particularly in older adults with atypical or nonspecific presentations [1, 9]. Clinical features may be variable, and classic findings such as fever may be absent, contributing to diagnostic uncertainty [9]. While delays in treatment are associated with worse neurological outcomes, meaningful clinical improvement may still occur following initiation of antiviral therapy, especially when treatment is ultimately administered during the active disease course [1]. This is reflected in our case, where antiviral therapy was initiated later in the hospital course, yet the patient demonstrated significant clinical improvement.

Atypical presentations of HSV‐1 encephalitis, with subtle or even normal neuroimaging or CSF profiles, further contribute to this delay in recognition and support the progressive, indolent nature of the disease in its early stages. It is important to note that imaging results suggestive of other or even concurrent conditions like NPH, for example, can mask distinct temporal lobe involvement that would strengthen an alternate diagnosis. Such ambiguity can delay consideration of infectious causes like HSV‐1. Early MRI findings may be limited, as several reports document cases of HSV‐1 encephalitis with minimal cingulate or insular abnormalities, followed by progression to more characteristic temporal lobe involvement [10]. This pattern emphasizes the limitations of relying solely on structural abnormalities or early neuroimaging findings. It reinforces the importance of maintaining a high degree of clinical suspicion even in the absence of definitive diagnostic results [1].

An important consideration is whether the patient’s initial presentation may have reflected a concurrent or suspected hydrocephalus‐related or neurodegenerative process, with HSV‐1 encephalitis evolving subsequently. Given the subacute progression of symptoms and initial imaging findings, this possibility cannot be entirely excluded. However, the patient’s rapid and marked clinical improvement following initiation of antiviral therapy strongly supports an infectious etiology as the primary driver of the neuropsychiatric presentation.

A growing body of evidence demonstrates that variability in the host immune response contributes to delayed recognition of HSV‐1 encephalitis. Although the disease typically produces a strong lymphocytic inflammatory profile, some patients, especially older adults, may exhibit minimal pleocytosis or clinical markers, making early presentations appear less overtly infectious and strengthening support for alternate differential diagnoses [1]. In this case, repeated lumbar puncture spinal taps failed to reveal significant pleocytosis, reinforcing a noninfectious etiology. Emerging immunologic studies further show that disruptions in TLR3 signaling, interferon pathways, and blood–brain barrier permeability can blunt early inflammatory responses despite ongoing viral replication, contributing to subtle but progressively worsening symptoms before detectable antigen levels [11]. These mechanisms help explain why clinical deterioration can precede confirmatory laboratory or imaging findings, reinforcing the importance of early recognition and timely intervention.

Timely initiation of antiviral therapy remains one of the strongest determinants of symptomatic and neurological recovery in patients with HSV‐1 encephalitis. Any delay in initiation of antiviral therapy, most commonly acyclovir, will allow for unchecked viral replication and increasing inflammation, both of which accelerate neuronal injury and worsen long‐term morbidity [8]. Several studies have demonstrated that initiating treatment more than 3 days after symptom onset significantly increases the likelihood of poor prognosis, severe neurological sequelae, and functional decline [12]. In this case, the patient remained hospitalized for several weeks without recognition of the underlying encephalitic process while being treated for symptoms of NPH and neurocognitive disturbances. Early diagnostic uncertainty commonly jeopardizes the narrow therapeutic window. While psychiatric consultation is not routinely required in typical cases of NPH, it may provide additional value in identifying atypical neurobehavioral features, particularly when the clinical presentation deviates from expected patterns or suggests an alternative underlying process. These findings reinforce the need to maintain a broad differential diagnosis and to initiate empiric antiviral therapy early in the evaluation process, as HSV‐1 encephalitis cannot be diagnosed with high certainty [8].

Diagnostic challenges and treatment delays may be further amplified in settings with limited access to advanced neuroimaging, specialty consultation, and rapid CSF analysis. This is especially relevant in rural settings where diagnostic delays are more common. Given the substantial impact of time‐to‐treatment on patient outcomes, these systematic scenarios highlight the importance of early empiric antiviral therapy, possibly without definitive serologic confirmation, and a high degree of clinical suspicion in patients presenting with unclear, progressive neurocognitive decline.

In this case, the psychiatric consultation service played a central role in reframing the patient’s initial diagnostic trajectory. Although their presentation initially aligned with NPH due to the gait instability, urinary incontinence, ventriculomegaly, and transient improvement after lumbar puncture, psychiatry ultimately noted several key behavioral and cognitive inconsistencies that were atypical for a purely structural etiology.

First, the patient exhibited fluctuating levels of consciousness, disorganized attention, and abrupt waxing and waning of orientation, suggestive of a delirium process rather than the more gradual, stable cognitive profile seen in NPH. Second, the patient’s well‐formed paranoid delusions, visual hallucinations, and sudden affective lability were not adequately explained by frontal–subcortical dysfunction alone. Instead, these symptoms were more consistent with limbic system involvement, particularly in the mesiotemporal regions, a hallmark of herpes simplex encephalitis. Psychiatry also recognized that the patient’s behavioral response to CSF drainage was inconsistent with true NPH. While gait improved transiently, the patient’s cognition and psychosis did not follow the typical pattern of “tap‐test responsiveness.” This dissociation raised suspicion that the structural findings represented central volume loss rather than true hydrocephalus.

Integrating these observations through a neuropsychiatric lens, the consultation team concluded that the clinical picture extended beyond a structural gait disorder. Psychiatry recommended expanding the diagnostic workup to include infectious, autoimmune, and inflammatory etiologies. This ultimately led to CSF PCR testing, which confirmed HSV‐1 encephalitis after nearly 3 weeks of diagnostic uncertainty. Once antiviral therapy began, the patient’s rapid improvement further validated psychiatry’s neurobehavioral reasoning.

Thus, psychiatric assessment played a decisive role by identifying inconsistencies and challenging the initial NPH diagnosis. Psychiatry’s ability to synthesize fluctuating mental status, psychosis phenomenology, and neuroanatomical correlations was essential in resolving the diagnostic puzzle and preventing further delay in definitive treatment. This case emphasizes the need for heightened clinical vigilance and swift diagnostic action in similar circumstances, as early recognition and treatment remain the most critical determinants of favorable symptomatic and neurological recovery in patients with HSV‐1 encephalitis.

## 4. Conclusion

This case highlights the diagnostic challenges of atypical HSV‐1 encephalitis, particularly when early imaging and clinical features mimic those of more common structural or neurodegenerative conditions, such as NPH. The patient’s prolonged hospitalization and delayed recognition of the viral etiology emphasize the need to maintain a broad differential in older adults with progressive cognitive or gait disturbances, even when CSF and neuroimaging results appear reassuring. Early empiric antiviral therapy remains critical, as clinical outcomes are closely tied to timely intervention. In resource‐limited or rural settings, where diagnostic delays are more likely, heightened vigilance and early consideration of HSV‐1 encephalitis are essential to improving neurological recovery and reducing preventable morbidity.

## Author Contributions

**Christopher E. Potts**: writing – original draft, writing – review and editing, conceptualization, investigation, revision. **Alana Kassis**: writing – original draft, writing – review and editing, conceptualization, investigation. **Akbar Ali**: writing – original draft, writing – review and editing, conceptualization, investigation. **James Issa**: writing – review and editing, investigation. **Usman Alizai**: writing – original draft, writing – review and editing. **Dakota May**: project administration, supervision, writing – review and editing, conceptualization.

## Funding

The authors received no specific grant or financial support from any public, commercial, or not‐for‐profit funding agency for the preparation of this case report.

## Disclosure

An abstract of this work was previously presented as a poster at the Association of Medicine and Psychiatry (AMP) 2025 Conference held on October 9, 2025 in Newport Beach, California.

## Ethics Statement

This single patient case report did not require Institutional Review Board approval in accordance with institutional policy. Written informed consent was obtained from the patient for publication of the case details and accompanying images. All identifying information has been removed, and the case has been described in a manner intended to protect patient privacy and confidentiality.

## Conflicts of Interest

The authors declare no conflicts of interest.

## Data Availability

The data that support the findings of this study are available upon request from the corresponding author. The data are not publicly available due to privacy or ethical restrictions.
